# Dilating Vascular Diseases: Pathophysiology and Clinical Aspects

**DOI:** 10.1155/2018/9024278

**Published:** 2018-08-26

**Authors:** Ertan Yetkin, Selcuk Ozturk

**Affiliations:** ^1^Private Yenisehir Hospital, Department of Cardiology, Mersin, Turkey; ^2^Ankara Education and Research Hospital, Department of Cardiology, Ankara, Turkey

## Abstract

Atherosclerotic disease of the vessels is a significant problem affecting mortality and morbidity all over the world. However, dilatation of the vessels either in the arterial system or in the venous territory is another vessel disease. Varicocele, pelvic, and peripheral varicose veins and hemorrhoids are aneurysms of the venous vascular regions and have been defined as dilating venous disease, recently. Coronary artery ectasia, intracranial aneurysm, and abdominal aortic aneurysm are examples of arterial dilating vascular diseases. Mostly, they have been defined as variants of atherosclerosis. Although there are some similarities in terms of pathogenesis, they are distinct from atherosclerotic disease of the vessels. In addition, pathophysiological and histological similarities and clinical coexistence of these diseases have been demonstrated both in the arterial and in the venous system. This situation underlies the thought that dilatation of the vessels in any vascular territory should be considered as a systemic vessel wall disease rather than being a local disease of any vessel. These patients should be evaluated for other dilating vascular diseases in a systematic manner.

## 1. Introduction

Atherosclerosis of the vessels has been one of the main causes of mortality in the world for decades and a major focus for basic and clinical investigation in cardiovascular era for more than a half century. It is a systemic disease with important sequels as a result of obstructive lesions in vascular regions distinct from heart, including brain, kidneys, mesentery, and limbs. The name of atherosclerosis directly calls the meaning of obstructive vascular disease in any vascular territory. The development of lipid-lowering, antithrombotic, thrombolytic, and catheter-based therapies has provoked considerable impact in reducing mortality and morbidity in terms of atherosclerotic burden or obstructive vascular disease [1]. Although dyslipidemia and hypertension have less prominent impact on peripheral vascular atherosclerotic disease, both coronary atherosclerosis and peripheral atherosclerotic disease share the same common major risk factors [1, 2].

In addition to atherosclerotic disease of the vessels, there are other vascular diseases named as arterial or venous aneurysm of different vascular territories, which have not been classified well enough in terms of pathophysiology. Coronary artery ectasia (CAE), intracranial aneurysm (ICA), and abdominal aortic aneurysm (AAA) are examples of arterial aneurysms [3]. Varicocele, pelvic, and peripheral varicose veins and hemorrhoids are aneurysms of the venous vascular system [4]. Recently, we have defined the term “dilating venous disease” for these venous aneurysms due to the fact that they share similar pathophysiological steps and their high coexistence at the clinical level [4]. Additionally, it is known from previous reports that there is high clinical coexistence of venous and arterial aneurysms [4–12].

Vascular dilatations show a diverse clinical spectrum as in obstructive counterpart depending on the regional circulation with different clinical manifestations and different prevalence. Pathophysiology of the vascular dilatations might show similarities or discrepancies with obstructive vascular disease but it is important to make a systematic approach in terms of vascular entities even the territories existing in different organs or system, such as gastrointestinal and genitourinary system. In this review, we would like to focus on the systemic insights into the dilating arterial diseases under the term of “dilating arterial disease”. Subsequently, we will put together all of these diseases under the name of “dilating vascular disease”.

## 2. Coronary Artery Ectasia

CAE is an angiographic definition of coronary artery pathology in which the diameter of the ectatic segment measures more than 1.5 times the diameter of an adjacent healthy reference segment [3, 30–32]. The main coronary angiographic characteristics of CAE are impaired coronary blood flow, delayed antegrade coronary dye filling, segmental back flow phenomenon (milking phenomenon), and stasis with local deposition of dye in dilated coronary segments [3, 30, 31]. The incidence of CAE ranges from 0.2% to 10% [33–36] with the largest* antemortem *series found in the Coronary Artery Surgery Study registry (4.9%) of 20087 patients referred for coronary angiography [31].

Histological examination has been performed in only a minority of studies [37, 38], typically revealing marked destruction and reduction of the medial elastic fibers with disruption of the internal and external elastic lamina, usually out of proportion to the degree of the intimal involvement. On the other hand, the loss of musculoelastic arterial wall components in CAE was noticed to be unrelated to local atheromatous burden [38, 39]. Although CAE has been known to be a variant of atherosclerosis in the literature, there are certain pathophysiologic mechanisms or clinical variables differentiating it from atherosclerosis [3, 40]. Furthermore, CAE has been supposed to be a local manifestation of systemic vessel wall abnormality. Functional loss of the musculoelastic components of the coronary artery media is considered to be the predominant aspect in the pathogenesis of CAE [3, 37, 39]. An ultrasonographic study has shown that patients with CAE coexisting with coronary artery disease (CAD) have a significantly lower carotid intima-media thickness compared to those with CAD and without CAE, indicating that the mechanism underlying CAE might differ from the ones observed in atherosclerosis [41]. Interestingly, decreased endothelium-independent vasodilatation has been shown in patients with CAE and CAD compared to those with CAD alone [42].

Increased nitric oxide (NO) exposure has also been implicated in the pathogenesis of aneurysm formation or CAE in the literature. A herbicide containing acetylcholinesterase inhibitor directly stimulates NO production by increasing acetylcholine [43, 44]. It has been proposed that chronic nitrite exposure may cause hyaline degeneration of the coronary artery intima-media resulting in abnormal coronary dilatation. Johanning et al. [45] and Fukuda et al. [46] have experimentally shown that NO production plays a major role in inflammation and aneurysm pathogenesis. Matrix metalloproteinases (MMPs), cysteines, and their inhibitors have all been shown to play a role in the pathogenesis of CAE [47–49]. Turhan et al. have reported also increased levels of C-reactive protein and adhesion molecules indicating an increased inflammatory process in patients with isolated CAE compared to both patients with and patients without CAD [50, 51].

Among the cardiovascular risk factors, age and presence of diabetes mellitus (DM) are inversely associated with the presence of CAE in contrast to obstructive CAD [52]. DM is a well-known risk factor positively associated with coronary atherosclerosis and its complications, i.e., cardiovascular events [53, 54]. Androulakis et al. [55] and Bermudez et al. [35] have reported significant independent and inverse association between CAE and DM. In accordance with these observations, increased prevalence of abdominal aortic aneurysm has been reported in patients without DM [56, 57].

## 3. Intracranial Aneurysm

ICA is another form of dilating vascular disease at the arterial site. ICAs are vascular abnormalities of the brain with a prevalence of 3.2% in the general population [58] and are commonly found at arterial junctions, bifurcations, or abrupt vascular angles where excessive hemodynamic stresses are exerted on arterial walls [59]. The association of ICAs with other vascular dilating diseases is weak in the literature. Norrgård et al. [60] had reported that only 0.6% of patients with ICA had associated AAA, and only 2.2% of patients with an AAA had associated intracranial aneurysms. A recent report by Miyazawa et al. [61], using more advanced diagnostic modalities, found that the incidence of this association was 7.2%.

The pathogenesis of ICA involves persistent pathological vascular remodeling with proteolysis/extracellular matrix degradation via MMPs and apoptosis with concomitant vessel wall inflammation [62–65]. There is a close relation between wall shear stress, endothelial dysfunction, and the downstream inflammatory reaction [66]. Cathepsin enzymes and their most abundant inhibitor cystatin C have also been implicated in the pathogenesis of ICAs. Dynamic changes in the media and eventual loss of this layer appear to contribute to aneurysm formation [67]. Aoki et al. have demonstrated increased expression of cathepsin B, cathepsin K, and cathepsin S in arterial wall of the cerebral aneurysms, but decreased expression of cystatin C [68]. Regarding the inhibitory effects of cystatin C on catabolic cathepsin enzymes, it is reasonable to expect increased vascular wall destruction leading to dilatation in involved segments [69].

Oxidative stress and NO have also been supposed to contribute to pathogenesis of vessel wall degradation in ICA through promotion of an inflammatory environment, alteration in flow hemodynamics, upregulation of smooth muscle cell phenotypic modulation and ultimately cell death, and induction of matrix remodeling [70]. Fukuda et al. [46] have demonstrated that NO, particularly that derived from inducible nitric oxide synthase (iNOS), is a key requirement for the development of cerebral aneurysm in an animal model study.

Major risk factors of atherosclerosis such as hypertension, smoking, and DM are insufficient to explain the pathogenesis of ICAs in terms of individual differences [71]. Multiple aneurysms are found in 15–45% of patients with ICA [72, 73], suggesting that a substantial number of patients are naturally aneurysm prone [74]. As in CAE and AAA, DM has not found to be a positive and independent factor for multiple ICA formation [75, 76]. Moreover, Gu et al. have pointed out that DM in elderly female patients might be a factor reducing subarachnoid hemorrhage [77].

## 4. Abdominal Aortic Aneurysm

The term “dilating arterial disease” was used by Martin et al. [78] in 1978. Then, Tilson and Dang reported dilatation of the iliac and suprarenal segments in a series of patients with AAA [79]. Ward et al. [80] have come to a conclusion, by showing increased diameter of carotid femoral and brachial artery diameter in patients with AAA, that there is a generalized “dilating diathesis” that may be unrelated to the atherosclerotic process. AAA has been shown to be independently associated with femoral and carotid artery diameter by Johnsen et al. [81]. Prevalence of AAA has been reported as 4.7% in men and 1.7% in women in a necropsy study of a population aged 56 to 74 years [82]. Thoracic aortic aneurysms seem to be much rarer, occurring in 4 to 5 of 1000 autopsy studies [83].

Increased prevalence of AAA in patients with pulmonary emphysema, inguinal hernia, and incisional hernia is the literature support to the pathophysiology of disease in regard to connective tissue disease [84–87]. An association between CAE and AAA has also been reported by Stajduhar et al. [9]. Of the 72 patients with AAA, 15 had CAE (20.8%). Sporadic coexistence of CAE and AAA has also been published in the literature [88, 89]. Popliteal artery aneurysm is the most common peripheral arterial aneurysm presenting bilaterally in 50% and coexisting with abdominal aortic aneurysm in 50% of the cases [90, 91].

From a histopathological point of view, tunica media layer has a pivotal role in the development of AAA as in ICA and CAE. Reduction of elastin (elastolysis), defects in collagen, cystic degeneration of the smooth muscle layer (media), and atherosclerosis are histopathological changes in AAA. This degeneration ultimately leads to widening of the vessel lumen and loss of structural integrity [92]. It is possible that larger persons may be at greater risk of aneurysm development because of these principles and the inherent weakness of the human abdominal aorta relative to other species. Generalized connective tissue disorder or being born with large and tortuous arteries prone to further dilatation has been raised as a possible mechanism of AAA [79].

Evidence indicates that oxidative stress within the aortic wall is closely involved in the pathogenesis of AAA. Oxidative stress facilitates leukocyte recruitment into the vasculature by modulating inflammatory cytokines [93]. In addition, reactive oxygen species alter the balance between destruction and regeneration of the aortic wall by enhancing matrix proteolysis through the upregulation of MMPs [94]. Guzik et al. [95] have shown that the NADPH oxidases, iNOS, and cyclooxygenases are predominant sources of superoxide anion in AAA. In animal model study, antioxidative treatment has decreased AAA incidence, inhibiting reactive oxygen species generation in aortic tissue during AAA development [96].

Many members of the cysteine, cathepsin, and MMP subfamilies are potent elastases and/or collagenases that mediate the degradation of these ECM proteins, leading to AAA expansion and rupture [97, 98]. Tissue inhibitor of MMP-1 (TIMP-1) plays a key role in preventing medial degradation through its ability to inhibit the MMPs involved in the disruption of the media [99, 100]. An imbalance in the MMP:TIMP activity ratio may underlie the pathogenesis of vascular diseases, such as AAAs. Cystein proteinases and cystatin C have been implicated in the pathogenesis of AAA formation and CAE. Shi et al. [101] have reported that cystatin C, the most abundant extracellular inhibitor of cysteine proteinases, is markedly reduced in human atherosclerotic and aneurysmal lesions. Furthermore, they found that the circulating levels of cystatin C are significantly lower in patients with dilated abdominal aortas, as determined by ultrasonography, compared with the levels in patients with a normal range of aortic diameter.

Although AAA and atherosclerosis share some common risk factors such as hypertension and smoking [56, 102, 103], presence of DM is a protective factor against AAA, in contrast to atherosclerosis [56]. Moreover, Blanchard et al. [56] have reported that neither clinical hypercholesterolemia nor serum levels of total cholesterol, low-density lipoprotein cholesterol, and high-density lipoprotein cholesterol are associated with AAA. In addition, the familial aggregation of AAAs suggests that genetic susceptibility may play a role in the pathogenesis [104–106].

## 5. Miscellaneous Vascular Dilatations

In the literature, there are numerous reports of vascular dilatations due to connective tissue disorders, inflammatory disorders, congenitally anomalies in other vascular territories, or localization apart from those we discussed above. Furthermore, genetically determined diseases or syndromes also constitute a considerable amount in clinical manifestation.

Azygos vein aneurysms are very rare causes of mediastinal masses and are usually described as accidental findings on chest roentgenogram [107]. Atrial septal aneurysm (ASA) can also be classified as congenital form of vascular dilatations either in right or in left atrium. Although it is not a truly tubular vascular aneurysm, ASA is worth citing in this section. It is defined as the protrusion of the atrial septum mainly at the fossa ovalis region more than 15 mm from the plane of atrial septum into right or left atrium [108]. Although the true prevalence of ASA in the adult population is not well known yet, in the literature prevalence ranges between 1 and 10% depending on the imaging method and patient selection [109–111]. Mugge et al. [109] have noted coexistence of mitral valve prolapses, tricuspid valve prolapses, Marfan's syndrome, aortic dissection, and sinus valsalva aneurysm in patients with ASA, supporting the concept of systemic inherent connective tissue abnormality as a cause. Moreover, valvular regurgitation and supraventricular arrhythmias are common concurrent pathologies in patients with ASA [111]. Increased prevalence of ASA has also been reported in Behçet's disease [112].

Behçet's disease is a syndrome consisting of aphthous stomatitis, genital ulceration, and uveitis triad first described by Hulusi Behçet in 1937 [113]. Behçet's disease is a type of vasculitis with a chronic and relapsing course, which affects arteries and veins of any size [13–16]. The pathogenesis of aneurismal complication is attributed to endarteritis of vasa vasorum which causes necrosis and thereby weakness in the vessel wall, leading to aneurysm formation. Cerebral, aortic, pulmonary, and coronary artery aneurysms have all been reported in Behçet's disease patients in the literature [14, 15, 17, 18].

Kawasaki disease is an acute systemic inflammatory illness of children, which can result in coronary artery aneurysms, myocardial infarction, and sudden death in healthy children. Clinical and epidemiologic features support an infectious cause, but the etiology remains unknown. Kawasaki disease is the most prevalent cause of acquired heart disease in children in developed countries. The best theory for Kawasaki disease etiology is that a ubiquitous infectious agent results in asymptomatic infection in most individuals but causes Kawasaki disease in a subset of genetically predisposed individuals [19–21].

Ehlers-Danlos syndrome is a heterogeneous group of connective tissue disorders caused by a deficiency in collagen synthesis and processing. The hypermobility type is the second most common variant of Ehlers-Danlos syndrome and can be associated with cardiovascular and gastrointestinal manifestations [22, 23].

Marfan syndrome is an autosomal dominant condition caused by mutations in* FBN1 *or* TGFBR2 gene regions*, occurs in 1 in 3000 to 1 in 10 000 live births, and affects the cardiovascular, skeletal, ocular, and pulmonary systems [24–26]. In patients with Marfan syndrome, aortic root dilatation is a common finding and can be a serious source of morbidity and mortality [25, 26].

Klippel-Trenaunay syndrome is a disease with a wide variety of manifestations depending on the type of vascular disorder and its location. Klippel-Trenaunay syndrome is characterized by the following triad of features: (1) cutaneous capillary malformations (usually port wine stains), which frequently are located laterally, need not extend over the entire affected limb, and may be found at sites other than the hypertrophied limb; (2) soft tissue or bony hypertrophy (or both); and (3) varicose veins or venous malformations, often with persistent lateral embryologic veins [27].

Servelle-Martorell syndrome is also known as phlebectatic osteohypoplastic angiodysplasia. It is characterized by limb hypertrophy caused by venous and rarely arterial malformations and skeletal hypoplasia [28, 29].

Miscellaneous diseases associated with dilating vascular diseases and localization of aneurysms are listed in Table 1.

## 6. Arterial versus Venous Dilatations

Arteries and veins resemble each other in that their walls contain three coats. The tunica intima (inner coat) comprises the endothelium, the adjacent basement membrane, the subendothelial connective tissue, and the internal elastic lamina. The tunica media (middle coat) is composed of smooth muscle cells, elastic lamellae including the external elastic lamina and collagen fibers. The tunica adventitia (outer coat) contains connective tissue, a few cells, macrophages, mast cells, fibroblasts, and the nerves and vessels that supply the vascular wall. Arteries are distinguished by an especially well developed muscle coat, which contains a varying amount of elastic fiber according to its site (predominantly elastic and muscular arteries). This layer is the driving force of the blood vessels by dilating (vasodilatation) and constricting (vasoconstriction) the diameter of the blood vessels; it regulates blood flow and blood pressure. Veins in general have wider lumen and thinner walls than arteries. The three coats are less well defined and the muscular coat is less well developed [114].

There are some common points both in arterial and venous dilating diseases that need to be discussed in this context. It is reasonable to suggest that the main underlying pathophysiological mechanism takes place mainly in the middle coat or layer of the vascular wall. Enzymatic activation, cellular changes, and biochemical processes degenerate the medial layer of the vessel, and subsequently vascular wall weakness occurs in all vascular regions. In addition, extracellular matrix remodeling by a serial enzymatic process including MMPs, serin proteinases, and cystein proteinases plays a role in both arterial and venous dilating diseases [3]. Similarly, oxidative stress and increased inflammation have all been documented in all entities. Increased NO stimulation or activity has been demonstrated to play a role in AAA, CAE, peripheral varicose vein, hemorrhoids, and even in pelvic congestion syndrome [45, 46, 93, 115–118]. Inhibition of NO stimulation has been shown to be protective against aneurysm formation. Possible hazardous effects of nitrate therapy should be taken into consideration in patients with CAE and in pelvic congestion syndrome patients.

**Figure 1:**
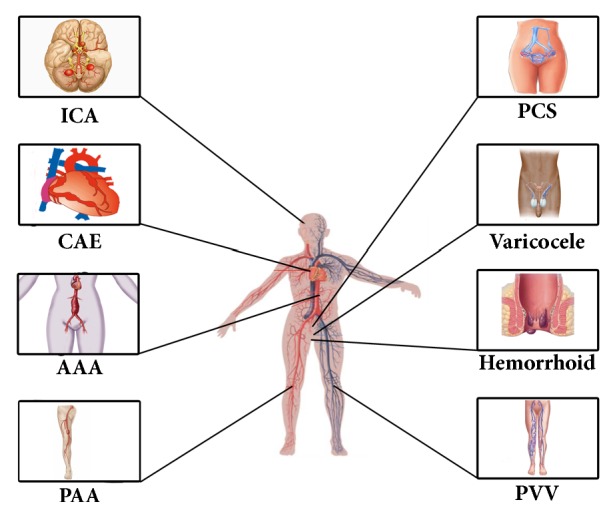
Arterial and venous dilating vascular diseases. AAA: abdominal aortic aneurysm, CAE: coronary artery ectasia, ICA: intracranial aneurysm, PAA: peripheral arterial aneurysm, PCS: pelvic congestion syndrome, PVV: peripheral varicose vein.

Among the risk factors, which have been shown to contribute to different vascular dilating disease processes, DM needs to be discussed as a separate entity. DM is well known and one of the most important cardiovascular risk factors positively associated with coronary atherosclerosis and its complications, i.e., cardiovascular events [53, 54]. Effects of DM on the progression of atherosclerosis have been shown by the demonstration of increased carotid artery intima-media thickness [3, 119]. A negative association of DM with arterial dilating vascular diseases has been reported in patients with CAE, AAA, and ICAs in different studies [52, 55, 56, 75, 77]. The prevalence of arterial and venous dilatations as a group seems to be different from each other. Arterial dilatations namely, CAE, AAA, and ICA, have quite similar ratio, roughly 1% to 5%. However, it should be noted that ratios might change depending on the method and definitions used in the studies. On the contrary, prevalence of venous dilatations, namely, peripheral varicose vein, varicocele, hemorrhoids, and pelvic congestion syndrome, is quite higher than those of arterial dilatations, having a larger range from 5% to 86%. This difference may be due to the contribution of physical forces in venous diseases such as gravitational force, height, constipation, occupation, and prolong standing [4]. In the arterial system, the effect of these forces on disease development is less defined. The main physical factor in the arterial system is the pumping force of the heart. It should also be kept in mind that venous dilating disease and its manifestations mainly occur below the heart level, in other words where the gravitational forces are more prominent. Arterial and venous dilating vascular diseases are depicted in Figure 1.

## 7. Conclusion

Coexistence of arterial aneurysm in different vascular territories, coexistence of venous dilatations in different vascular territories, and coexistence of arterial and venous dilations strongly suggest that both arterial and venous dilating disease arise from a common vascular wall pathology and need to be evaluated under the term of “dilating vascular disease”. Depending on where the vascular territory lies, different contributing factors play a role in the clinical manifestation of the disease. In the light of these similarities and coexistence demonstrated in the literature, we suggest that every patient with any detected dilating vascular disease should be systematically evaluated for other dilating vascular diseases. However, it is certain that to understand the pathophysiology and to develop new treatment modalities for the formation and progression of the disease, there is a need for further studies.

## Figures and Tables

**Table 1:** Miscellaneous diseases associated with dilating vascular diseases.

**Disease **	**Location of aneurysm**	**References**
Behçet's disease	Aortic aneurysm, pulmonary aneurysm, cerebral aneurysm, coronary aneurysm	[13–18]
Kawasaki disease	Coronary aneurysm	[19–21]
Ehlers-Danlos Syndrome	Peripheral Varicose veins, abdominal aorta aneurysm	[22, 23]
Marfan Syndrome	Aortic aneurysm	[24–26]
Klippel-Trenaunay syndrome	Peripheral Varicose veins	[27]
Servelle-Martorell syndrome	Venous aneurysm	[28, 29]
